# Isolation and Characterization of the Chromatic-Acclimating, Filamentous Cyanobacterium *Pseudanabaena* sp. Strain SR411

**DOI:** 10.17912/micropub.biology.001280

**Published:** 2024-08-22

**Authors:** Emma Hundermark, Emily Stowe

**Affiliations:** 1 Biology Department, Bucknell University

## Abstract

We isolated *Pseudanabaena* sp. Strain SR411, a novel filamentous, nonheterocystous, freshwater cyanobacterium from the West Branch of the Susquehanna River in Pennsylvania. Analysis of phycobilisome protein accumulation indicates *Pseudanabaena* SR411 acclimates to changing light wavelengths and we classified it as a chromatic acclimating cyanobacterium type CA3. The 5,780,083 bp genome has a GC content of 42.2% in which we identified 5,218 coding sequences and 58 RNA sequences. The genome includes putative homologs to the CA3 regulatory proteins RcaE, RcaF and RcaC.

**Figure 1. Light conditions alter the phycobiliprotein composition of the phycobilisome in Pseudanabaena SR411:**
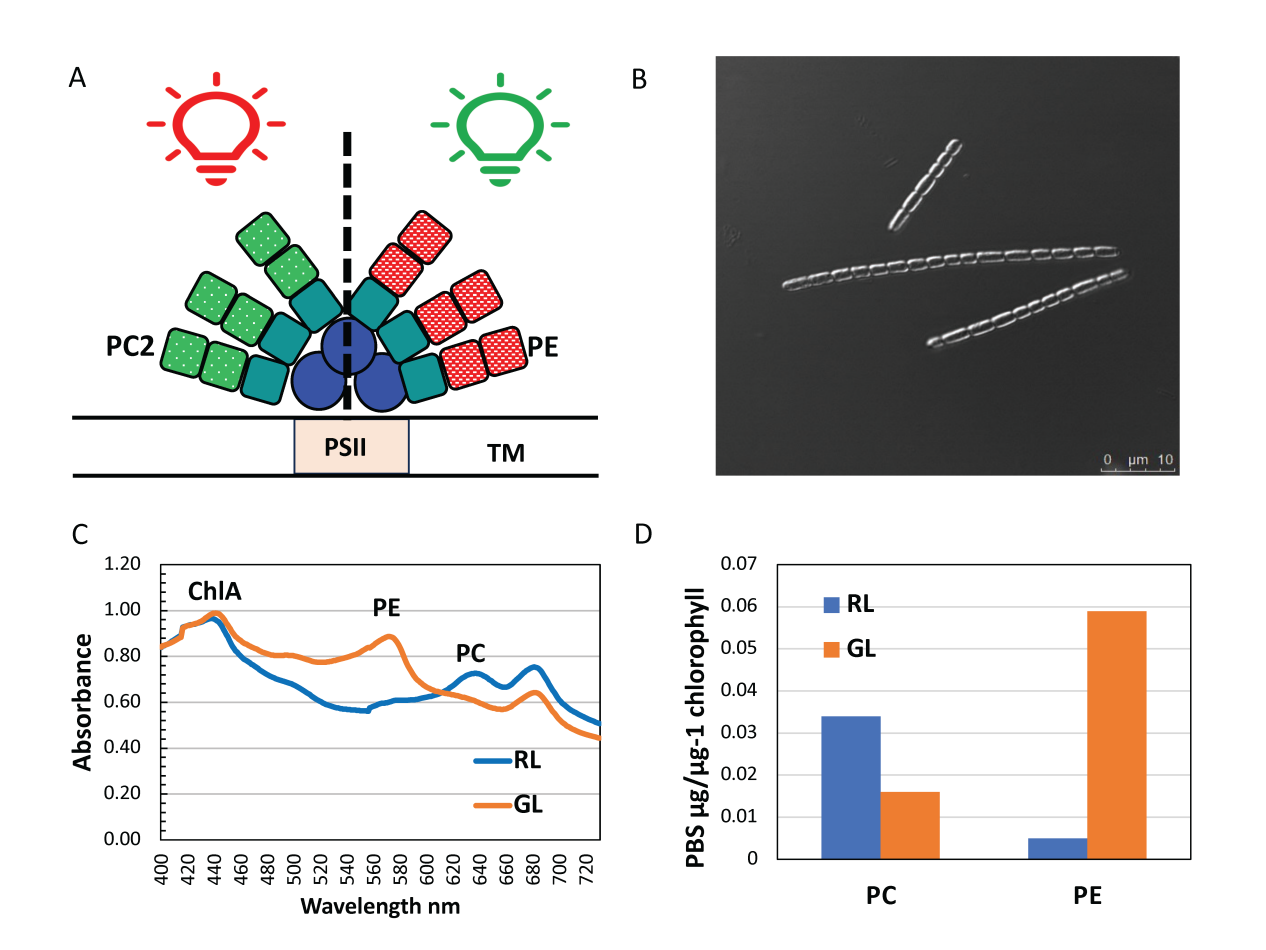
A. The distal proteins in the phycobilisome (PBS) of chromatic acclimating (CA) cyanobacteria vary in red light (RL) and green light (GL). In all light conditions, the core of the PBS contains allophycocyanin (AP, λ _max_ = 652nm, blue circles) and an inner layer of phycocyanin (PC, λ _max_ = 620nm, turquoise squares) (Adir, 2005). The distal layer of phycobiliproteins contains either PC (green dotted squares or phycoerythrin (PE, λ _max_ = 560nm, red checked squares) depending on the environmental light conditions (Adir, 2005). PSII: photosystem 2, TM: thylakoid membrane B. Confocal image of a filament from *Pseudanabaena* SR411 grown in RL. Unlike some CA species (Bennet and Bogorad, 1973, Singh and Montgomery, 2014), cell shape of *Pseudanabaena* SR411 does not alter when grown in GL. C. Whole cell absorbance spectrum from cells grown in RL and GL. In GL grown cells, PE is abundant and PC less abundant, whereas in RL PC is very abundant and PE is not detectable. D. PBS protein measurements from cells grown in RL and GL. Measurement of isolated PBS proteins again indicate that PE is more abundant in GL than RL and PC levels decrease in GL compared to RL. These data support the conclusion that *Pseudanabaena* SR411 is a CA3 species (Sanfilippo et al., 2019).

## Description

As photosynthetic bacteria, cyanobacteria make important contributions to both the carbon and nitrogen cycles (Whitton and Potts, 2000) . In order to maintain photosynthetic efficiency in a changeable light environment, many cyanobacteria produce phycobilisomes (PBS) specifically tuned to the light conditions (Green 2007; Figure 1A ). Chromatic acclimation (CA) is used to produce red light (RL) absorbing PBS containing phycocyanin (PC) or green light (GL) absorbing PBS containing phycoerythrin (PE, Bennet & Bogorad, 1973, Kehoe and Gutu, 2006). Seven forms of CA have been described that allow cyanobacteria to respond to changes in light conditions (CA1-7, Sanfilippo et al., 2019). CA1-3 processes alter the PE and PC contents of the PBS in response to light color. Specifically in CA3, the terminal rod components are PC in RL but PE in GL (Tandeau de Marsac 1977, Sanfilippo et al., 2019) . CA optimizes light absorption for photosynthesis by altering PBS composition in fluctuating light environments and in environments where competition for specific wavelengths might exist (Stomp et al., 2004, Stomp et al., 2007) . In a shallow river, like the Susquehanna River, attenuation of RL is not expected and therefore CA species might not be favored or expected to be abundant. However, a competitive light environment could favor CA species (Stomp et al., 2004, Stomp et al., 2007) . We thus began to explore the diversity of cyanobacteria in the Susquehanna River to determine if CA species existed in this environment. Preliminary analysis of this unpublished metagenomic data indicates that there are at least three genera with known CA species in the river: *Calothrix* , *Nostoc* and *Pseudanabaena* (Wang et al., 2022) . We isolated a novel strain named *Pseudanabaena* SR411 from surface water samples of the Susquehanna River. The cells of *Pseudanabaena* SR411 are rod shaped with rounded edges ( Figure 1B ). *Pseudanabaena* SR411 has sheath-less filaments growing in a single plane, does not form heterocysts or akinetes. These characters place it in Section III of the major groups of cyanobacteria (Rippka et al., 1979) . The 16S rRNA gene was amplified (Weisburg et al., 1991) and analyzed on EZBioCloud where it showed 99.43% identity to *Oscillatoria limnetica* MR1 (also known as *Pseudanabaena limnetica* ) and 99.36% identity to *Pseudanabaena biceps* (Yoon et al., 2017) . Whole cell spectra analysis indicates that PE is nearly absent in RL grown cells while PC is reduced in GL grown cells compared to RL grown cells ( Figure 1C ). Subsequent measurement of isolated phycobiliproteins indicated that PC is 2 fold more abundant in RL compared to GL and PE is 11 fold more abundant in GL compared to RL ( Figure 1D ). This allows us to classify the CA response in *Pseudanabaena* SR411 as CA3. We will confirm this observation via qPCR analysis of PBS genes which will allow us to determine which of the two identified PC gene encoding operons is RL inducible (PC2) and which is constitutive (PC1). The draft genome assembly of *Pseudanabaena* SR411 consists of 264 contigs, totaling 5,780,083 base pairs, with a GC content of 42.2%; the final coverage is 16X and N50 is 49,591. The genome size falls within the expected range for *Pseudanabaena* species (2.5Mbp-13.6Mbp) (Herdman et. al., 1979, Chen et al., 2021) , but is smaller than other CA cyanobacteria such as *Nostoc punctiforme* (CA2) (Meeks et al., 2001) and *Tolypothrix* sp. PCC 7601 (CA3) (Yerrapragada et al., 2015) . CA1-3 have been confirmed in other *Pseudanabaena* species (Wang and Chen, 2022, Su et al., 2023) . As expected for a CA3 organism, multiple phycocyanin operons, phycoerythrin, associated bilin synthases and lyases were identified. Additionally, we identified putative homologs of CA3 regulatory proteins RcaE (WP_094535076), RcaF (WP_094535075) and RcaC (WP_094535074), which have 72%, 87.9% and 59.6% identity, respectively, to the homologs in the model organism *Tolypothrix* PCC7601 (Kehoe and Grossman, 1997, Terauchi et al., 2004, Kehoe and Gutu, 2006) . These regulatory genes are located within close proximity to the PE encoding structural genes and both PC and PE bilin synthase genes. The presence of these regulatory genes further supports classifying *Pseudanabaena* SR411 as a CA3 organism. While we identified *nifHDK* homologs, indicating the potential to fix nitrogen (Tsygankov, 2007) , we have not yet found conditions in which nitrogen fixation is induced. *Pseudanabaena* SR411 will provide information useful in studying the maintenance of photosynthetic efficiency in fluctuating and competitive light conditions and the regulation of nitrogen fixation in nonheterocystous cyanobacteria.

## Methods

*Pseudanabaena* SR411 was isolated from surface water of the Susquehanna River collected April 2011 in Lewisburg, Pennsylvania (40.9645° N, 76.8844° W). The strain was isolated on BG11 media supplemented with 10 mM HEPES (pH 8.0) and 1.5% w/v agar in white light and subsequently grown in liquid BG11 in red or green light and bubbled with air (for conditions see Stowe et al., 2011). Unialgal cultures were obtained by repeated streaking on BG11. This strain can be obtained by communicating with the corresponding author. We characterized the strain using a combination of physical and genetic characteristics. *Pseudanabaena* SR411 has a straight trichome, with cylindrical cells, moderate constrictions between cells, no sheath, and cells with more rounded edges (Fig 1B). *Pseudanabaena* SR411 exhibits filamentous growth in one plane and reproduce through trichome breakage. This strain did not produce heterocysts when grown in a low nitrogen environment. Using the morphologically-based classification rubric devised by Rippka et al. (1979), these characteristics place *Pseudanabaena* SR411 in subsection III of the major groups of cyanobacteria. Whole cell and isolated PBS composition was done on fully adapted cultures brought to equivalent concentration by *A*_750_ measurement on a Beckman DU640 spectrophotometer as described in Stowe et al. (2011). We isolated DNA from RL grown cells using a ZR Fungal/Bacterial DNA MiniPrep™ kit (Zymo Research D4068) and the genome was sequenced at Genomic Services Lab at HudsonAlpha Institute for Biotechnology ( https://www.hudsonalpha.org/gsc/capabilities/ ) using an Illumina HiSeqX 150PE platform. We assembled the genome using NextGENe v2.2.0 software from SoftGenetics® using default parameters ( https://softgenetics.com/products/nextgene ). Annotation by NCBI Prokaryotic Genome Annotation Pipeline 4.1 identified 5218 coding sequences and 58 RNA genes (Tatusova et al., 2016) . This organism was submitted to NCBI as BioSample SAMN06761459 and BioProject PRJNA383344 and assembly GCA_002251945.1 . Genome Assembly accession number ASM225194v1 . This Whole Genome Shotgun project was deposited at DDBJ/EMBL/GenBank as accession NDHW01 .
